# Supplementary material for: Management, Analyses, and Distribution of the MaizeCODE Data on the Cloud
Source: Front Plant Sci. 2020 Mar 31;11:289. doi: 10.3389/fpls.2020.00289 (PMC7136414; doi:10.3389/fpls.2020.00289)
Supplement: Supplementary file 1 [file Data_Sheet_1.docx]

**Supplementary Materials for**

**Management, Analyses, and Distribution of the MaizeCODE Data on the Cloud**

Liya Wang1, Zhenyuan Lu1, Melissa delaBastide1, Peter Van Buren1, Xiaofei Wang1, Cornel Ghiban1, Michael Regulski1, Jorg Drenkow1, Xiaosa Xu1, Carlos Ortiz-Ramirez2, Cristina Fernandez-Marco1, Sara Goodwin1, Alexander Dobin1, Kenneth D. Birnbaum2, David P. Jackson1, Robert A. Martienssen1, William R. McCombie1, David A. Micklos1, Michael C. Schatz1,3, Doreen H. Ware1,4,*, Thomas R. Gingeras1,*

1Cold Spring Harbor Laboratory, Cold Spring Harbor, NY, 2New York University, New York, NY, 3Johns Hopkins University, Baltimore, MD. 4USDA-ARS Robert W. Holley Center for Agriculture and Health, Ithaca, NY, United States

**1. List of updates to the platform since the SciApps Bioinformatics paper (May 2018)**

- June 2018: Added job submission retrying to deal with underline AGAVE API glitches
- July 2018: Added user workflow management page, including Relaunch, Visualize, Load, Share and Delete
- Aug. 2018: Added user job management page, including Load, Refresh, and Delete
- Sept. 2018: Added matched job number from History panel to the workflow diagram
- Sept. 2018: Added option for redrawing workflow diagram in ‘Top-Down’
- Sept. 2018: Added calculating workflow depth for optimizing diagram display
- Sept. 2018: Added visualization button in workflow page for the workflow diagram
- Oct. 2018: Added support for apps with a variable number of inputs
- Nov. 2018: Added curved links in the workflow diagram
- Dec. 2018: Initialized RESTful API development for MaizeCODE DCC
- Mar. 2019: Enabled copying Genome browser links and workflows links to the clipboard
- April 2019: Finalized API development
- April 2019: Supported compression/decompression of the files to be visualized
- April 2019: Built Genome service on TACC cluster to support processing MaizeCODE data
- April 2019: Added Data page for the MaizeCODE DCC
- May 2019: Build API for workflow building with certain steps replaced (not done with the JavaScript code)
- May 2019: Redesigned ‘Load Workflow’ to allow loading more than one workflow (e.g., to support differential expression analysis between two MaizeCODE tissues)
- June 2019: Associated metadata to workflows and supported metadata retrieval
- July 2019: Bug fixes due to underline API changes (from Agave to Aloe)
- Aug. 2019: Separated loading job histories from relaunching workflows
- Sep. 2019: Developed API for updating a workflow with a new workflow ID

**2. Example commands for calling SciApps RESTful API**

1. Retrieving a token from SciApps (needs permission)
   - token="Authorization: Bearer 77f32c7173ebe6578df669ca449b0”
2. Submitting a workflow using a template workflow with replacement
   - curl -X POST -sk -H "user: lwang" -H "$token" https://www.sciapps.org/workflowJob/new?runWorkflowJob=1 -F fileToUpload=@test.json
   - Content of test.json

{

"wf_step_0:trait": "agave://data.iplantcollaborative.org/lwang/sci_data/height.txt",

"_derived_from": "5fb15c8f-0d38-4ec3-9b0e-392b59f08883"

}

- - “_derived_from” allows looping through MaizeCODE experiments in a batch mode (using a template workflow with changing input files)

1. Submitting a job
   - curl -X POST -sk -H "user: lwang" -H "$token" https://www.sciapps.org/job/new/ConvertTraitID-0.0.3 -F fileToUpload=@test.json
   - Content of test.json

{

"sid": "Sorghum",

"header": 0,

"trait": "agave://data.iplantcollaborative.org/lwang/sci_data/height.txt"

}


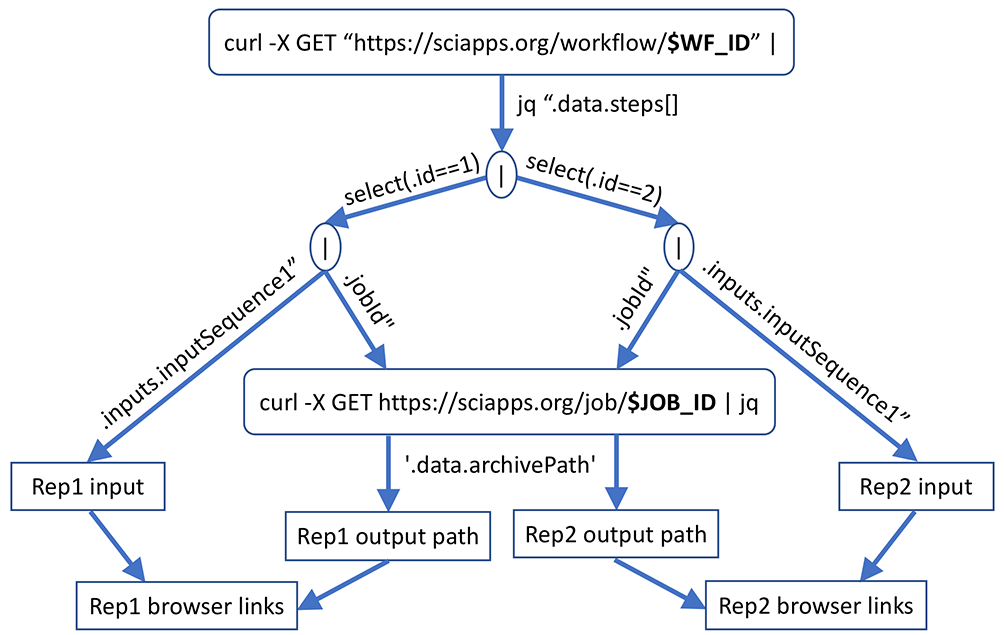


**Figure S1. Flowchart of generating genome browser tracks via cURL** **for a MaizeCODE workflow**. Curl is a free and open-source software used in command lines or scripts to transfer data. Each workflow has two replicates. **$WF_ID** is the workflow ID, and **$JOB_ID** is the job ID of a step of the workflow. As an example, the cURL for retrieving the input filename of replicate 1 is: curl -X GET --header "Accept: application/json" "https://www.sciapps.org/workflow/$WF_ID" | jq ".data.steps[] | select(.id==1) | .inputs.inputSequence1". Where **jq** is a lightweight and flexible command-line JSON processor. **jq** is like sed for JSON data – you can use it to slice and filter and map and transform structured data with the same ease that sed, awk, and grep let you play with text.


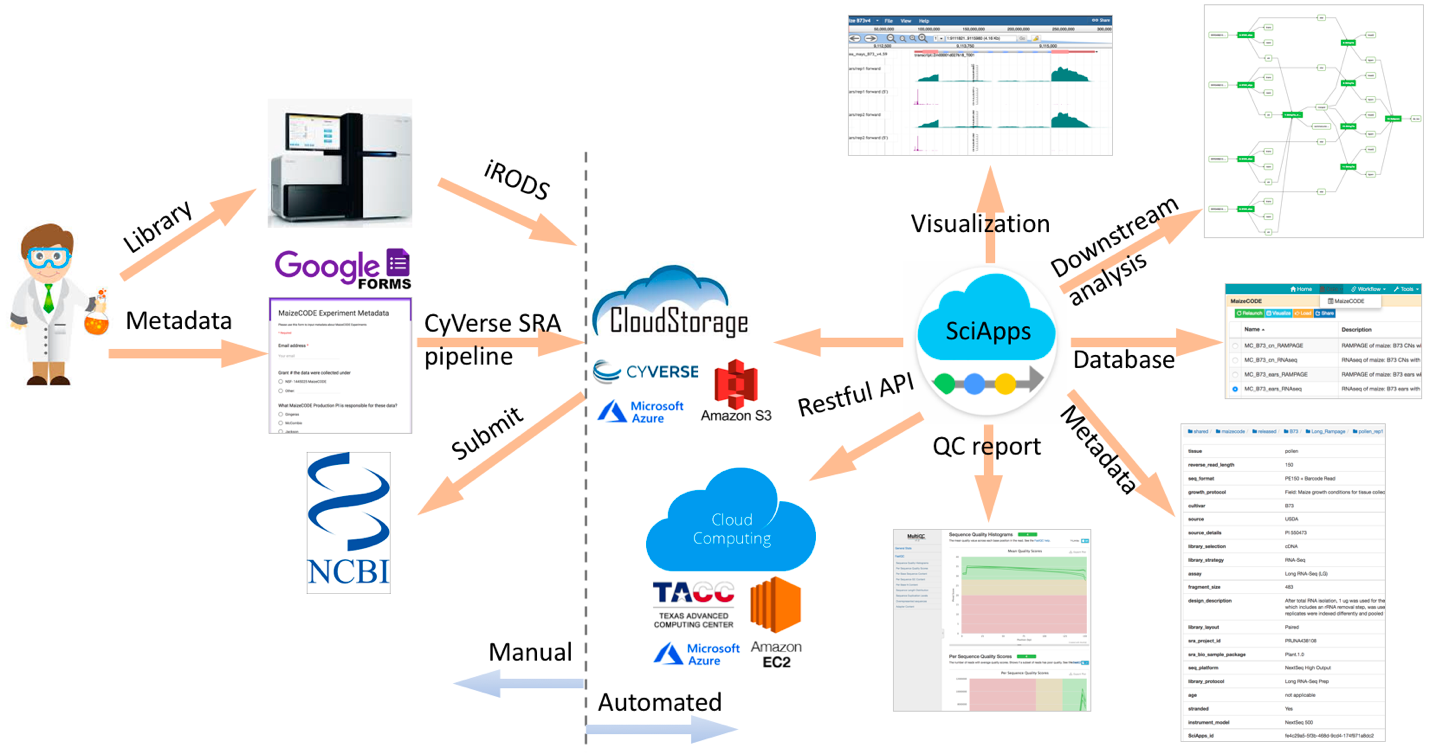


**Figure S2. Overview of MaizeCODE data flow, management, processing, and releasing**. From left to right: Sequencing libraries are prepared by MaizeCODE scientists and sent to sequencing center; Raw reads are deposited into the CyVerse Data Store; Metadata is gathered with predefined metadata forms and attached to the raw data by following the NCBI SRA requirement; Data are validated by the MaizeCODE group then submitted to NCBI SRA; SciApps workflows are used to bring the raw data from CyVerse Data Store to TACC for computing and results are archived back to the CyVerse Data Store (batch processed via API). MaizeCODE data sets and analyses are organized as automatic workflows in the database; Genome browser tracks are automatically generated via API; QC report, metadata, and downstream analysis are supported once workflows are loaded via unique IDs.


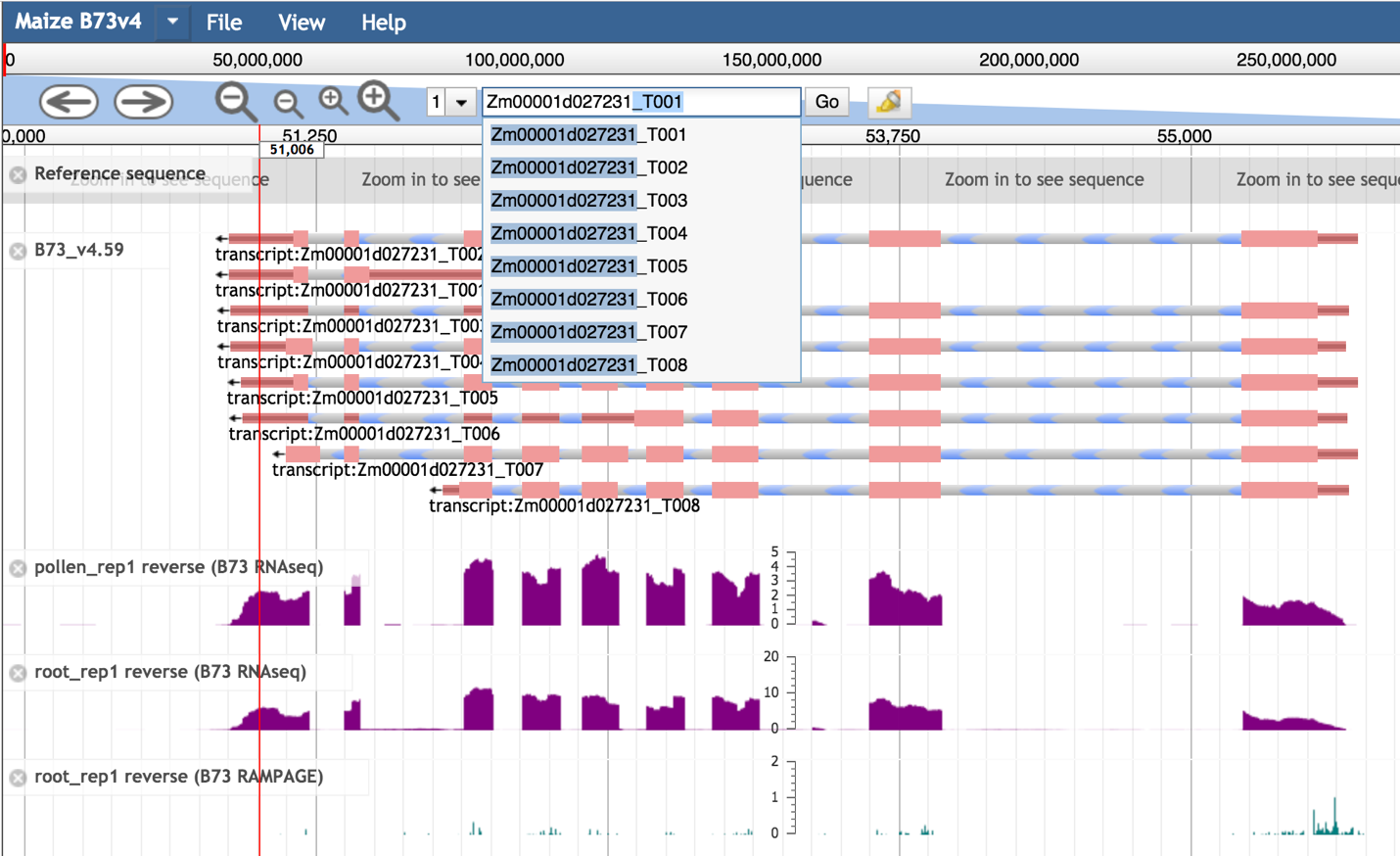


**Figure S3. Example of searching with gene ID in JBrowse**. After pasting the gene ID into the location text box, users will be prompted to select from a list of transcripts (isoforms). Make the selection, and click ‘Go’, the browser will zoom into the selected gene region.
